# Supplementary material for: The interactome of intact mitochondria by cross-linking mass spectrometry provides evidence for coexisting respiratory supercomplexes
Source: Mol Cell Proteomics. 2017 Dec 8;17(2):216–32. doi: 10.1074/mcp.RA117.000470 (PMC5795388; doi:10.1074/mcp.RA117.000470)
Supplement: Supplemental Data [file supp_RA117.000470_133922_0_supp_23978_fzffwf.zip › spectra_annotation/mito_DR_spectra_annotation/307-1-36-1-7-1.pdf]

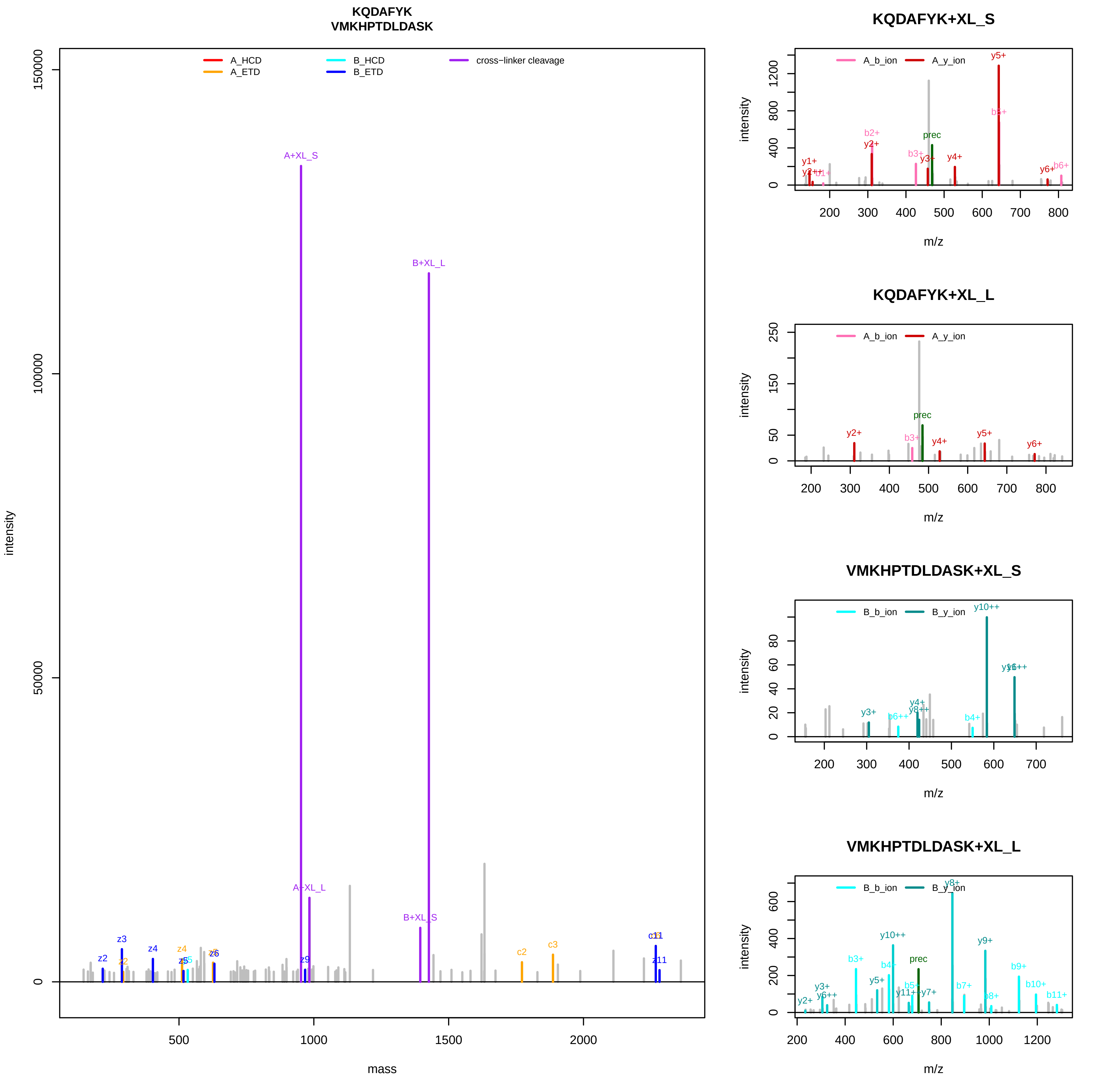

KQDAFYK
VMKHPTDLDASK
A_HCD
A_ETD
B_HCD
B_ETD
cross−linker cleavage
A+XL_S
B+XL_L
A+XL_L
B+XL_S
z2
z3
z4
z5
z6
z9
z11
c2
c3
c11
intensity
mass
KQDAFYK+XL_S
A_b_ion
A_y_ion
y5+
b2+
y2+
prec
b3+
y3+
y4+
y1+
y6+
b6+
KQDAFYK+XL_L
VMKHPTDLDASK+XL_S
B_b_ion
B_y_ion
y10++
y3+
b4+
VMKHPTDLDASK+XL_L
y8+
y9+
b3+
b9+
b10+
b11+
b7+
y5+
y7+
y2+
m/z
